# Supplementary material for: Detrimental proarrhythmogenic interaction of Ca2+/calmodulin-dependent protein kinase II and NaV1.8 in heart failure
Source: Nat Commun. 2021 Nov 15;12:6586. doi: 10.1038/s41467-021-26690-1 (PMC8593192; doi:10.1038/s41467-021-26690-1)
Supplement: Supplementary file 1 — Supplementary information [file 41467_2021_26690_MOESM1_ESM.pdf]

Supplementary Information for

Detrimental proarrhythmogenic interaction of Ca^{2+}/calmodulin-dependent protein kinase II and $Na_V1.8$ in heart failure

Philipp Bengel*, Nataliya Dybkova*, Petros Tirilomis*, Shakil Ahmad, Nico Hartmann, Belal Mohamed, Miriam Celine Krekeler, Wiebke Maurer, Steffen Pabel, Maximilian Trum, Julian Mustroph, Jan Gummert, Hendrik Milting, Stefan Wagner, Senka Ljubojevic-Holzer, Karl Toischer, Lars S Maier, Gerd Hasenfuss, Katrin Streckfuß-Bömeke*, Samuel Sossalla*†

†Corresponding Author: Email: samuel.sossalla@klinik.uni-regensburg.de

The file includes:

Supplementary Fig. 1: Comparison of *SCN5A* and *SCN10A* mRNA levels in human myocardium and isolated cardiomyocytes

Supplementary Fig. 2: *SCN10A* mRNA expression in myocardial tissue and isolated cardiomyocytes from WT and CaMKIIδc$^{+/T}$ mice

Supplementary Fig. 3: Current-voltage relationship of I_{NaL} in cardiomyocytes from CaMKIIδc$^{+/T}$ mice treated with vehicle or PF-01247324

Supplementary Fig. 4: Influence of $Na_V1.8$ knock-out on peak I_{Na} magnitude and current kinetics

Supplementary Fig. 5: CRISPR/Cas9 generated homozygous *SCN10A* knock-out iPSC-lines and cardiac differentiated iPSC-cardiomyocytes

Supplementary Fig. 6: Influence of $Na_V1.8$ inhibition on Ca^{2+}-transient kinetics in Wild-Type (WT) mouse cardiomyocytes

Supplementary Fig. 7: Influence of $Na_V1.8$ knock-out on $Na_v1.5$ protein and mRNA expression in mice

Supplementary Fig. 8: Influence of $Na_V1.8$ knock-out on CaMKIIδc protein and mRNA expression in mice

Supplementary Fig. 9: Ca^{2+}-transient amplitude and kinetics in cardiomyocytes from CaMKIIδc$^{+/T}$ and SCN10A$^{-/-}$/CaMKIIδc$^{+/T}$ mice

Supplementary Fig. 10: Amino acid sequence comparison of the I-II intracellular loop of human $Na_V1.5$ and $Na_V1.8$

Supplementary Fig. 11: Scheme of crossbreeding of SCN10A$^{-/-}$ and CaMKIIδc$^{+/T}$ mice

Supplementary table 1: Action potential characteristics of ventricular cardiomyocytes from WT, SCN10A$^{-/-}$, CaMKIIδc$^{+/T}$ and SCN10A$^{-/-}$/CaMKIIδc$^{+/T}$ mice

Supplementary table 2: Baseline ECG-parameters of CaMKIIδc$^{+/T}$ and SCN10A$^{-/-}$/CaMKIIδc$^{+/T}$ mice

Supplementary table 3: Patient characteristics of human failing hearts used for experiments

Supplementary table 4: List of all primers used in this study

Supplementary Figures:

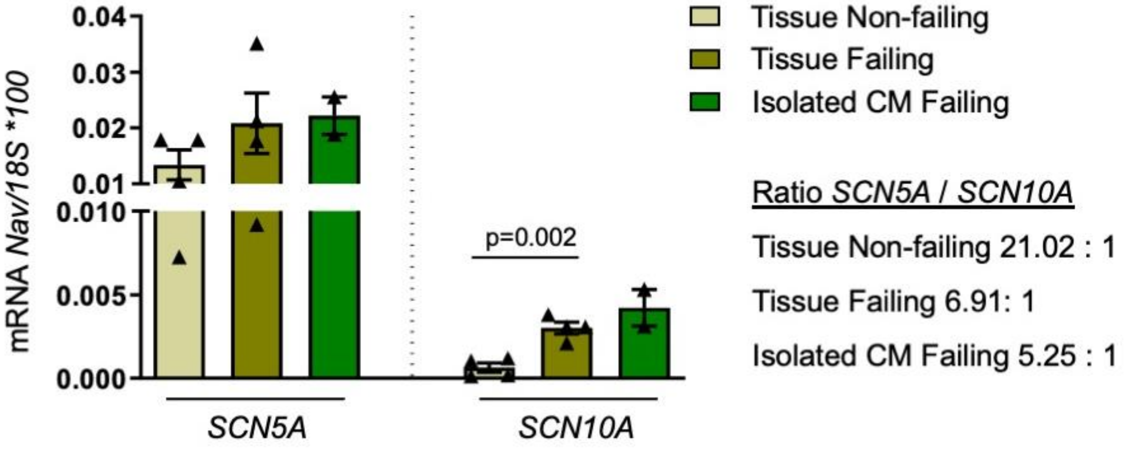

Supplementary Fig. 1: Comparison of *SCN5A* and *SCN10A* mRNA levels in human myocardium and isolated cardiomyocytes.

Comparison of *SCN5A* and *SCN10A* mRNA levels in tissue from non-failing and failing hearts, as well as isolated cardiomyocytes (CM) from failing hearts, RT-qPCR showing up-regulation of *SCN10A* mRNA in human HF ventricular myocardium (p=0.002 vs NF, unpaired two-tailed Student's test) and its presence as mRNA in isolated cardiomyocytes from human HF (NF: n=4, HF: n=4, CM n=2). Data are presented as mean values ± SEM.

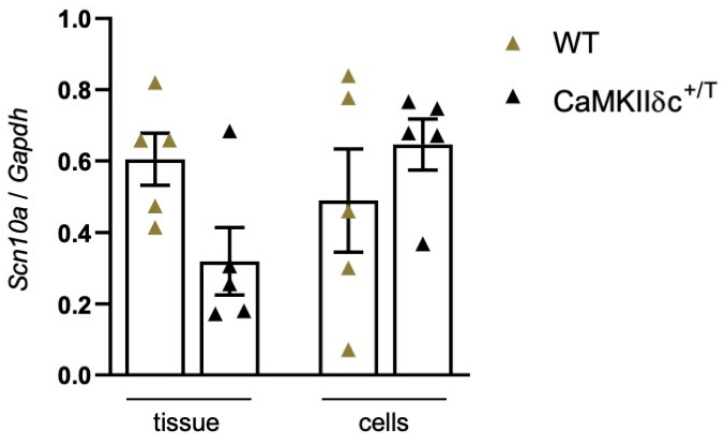

Supplementary Fig. 2: *Scn10a* mRNA expression in myocardial tissue and isolated cardiomyocytes from WT and CaMKIIδc$^{+/T}$ mice

A relevant amount of *Scn10a* mRNA in murine myocardium originates from cardiomyocytes (tissue: Wild-Type (WT): n= 5 mice, CaMKIIδc$^{+/T}$: n=5 mice; cells: WT: n= 5, CaMKIIδc$^{+/T}$: n=5). Data are presented as mean values ± SEM.

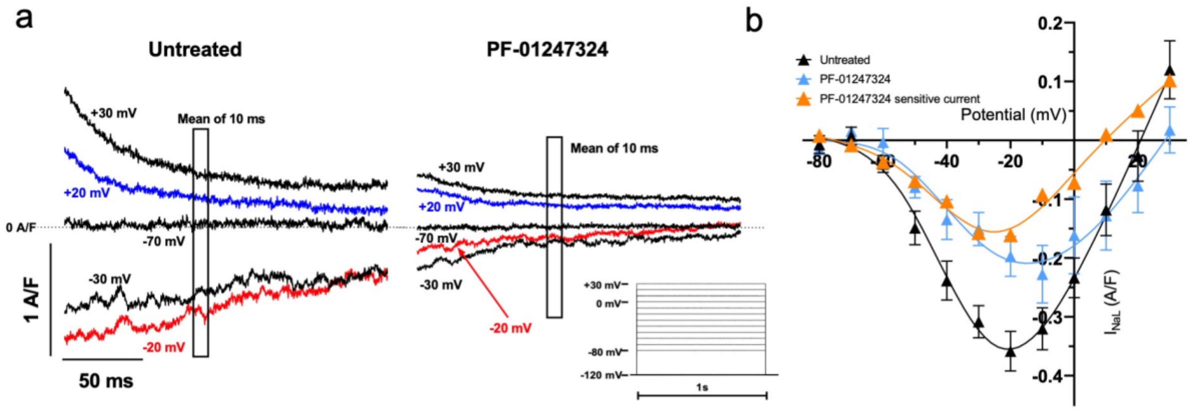

Supplementary Fig. 3: Current-voltage relationship of I_{NaL} in cardiomyocytes from CaMKIIδc$^{+/T}$ mice treated with vehicle or PF-01247324

a Original traces of the current response between 100 and 300 ms of the respective voltage step in vehicle- and PF-01247324-treated ventricular cardiomyocytes elicited by application of the voltage step protocol shown in the inset. The box depicts the time frame between 180 and 190 ms used for analysis of I_{NaL}.

b I-V curves (Mean data±SEM) of I_{NaL} in ventricular cardiomyocytes from CaMKIIδc$^{+/T}$ mice treated with vehicle or PF-01247324. I_{NaL} densities are significantly reduced in PF-01247324-treated (n=21 cells/9 mice) compared to vehicle-treated (n=13 cells/6 mice) ventricular cardiomyocytes at -30 mV (p=0.004) and -20 mV (p=0.023, mixed effects analysis with Holm-Sidak´s post-hoc test). PF-01247324 sensitive I_{NaL} was calculated as the difference between the mean I_{NaL} densities of vehicle and PF-treated cardiomyocytes at each membrane potential.

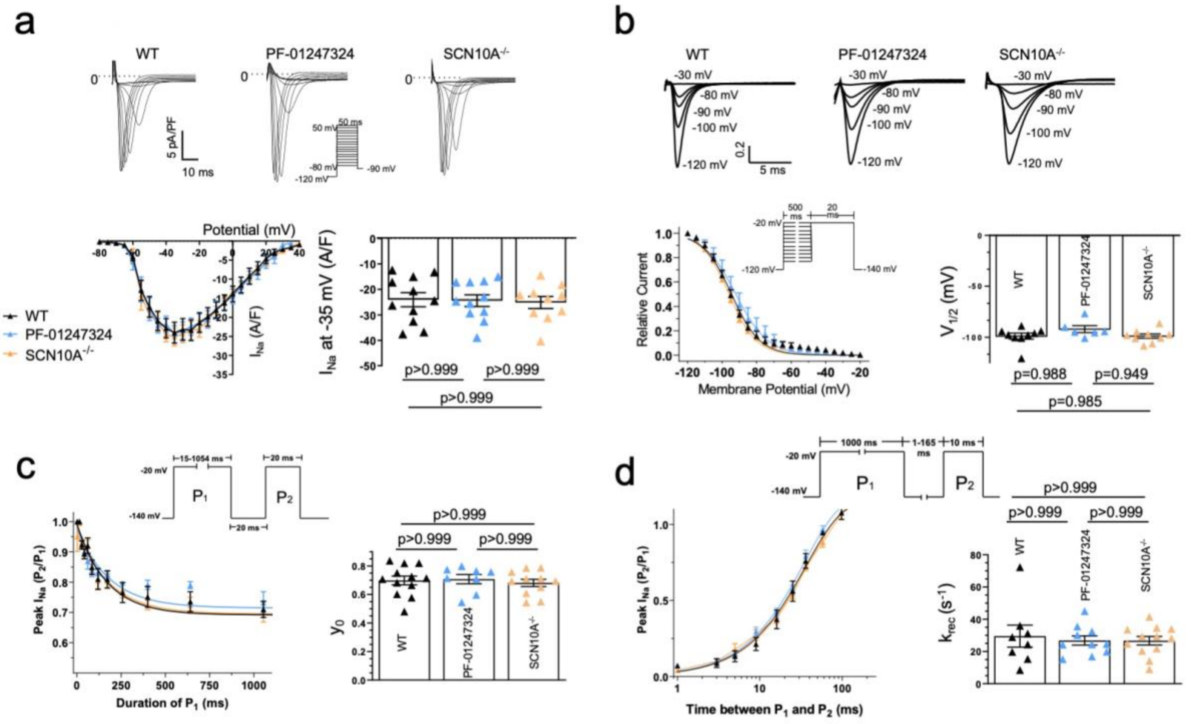

Supplementary Fig. 4: Influence of Na_V1.8 knock-out on peak I_{Na} magnitude and current kinetics

a Original traces and average current-voltage (I-V) relationships (measured with the protocol in inset) in mouse cardiomyocytes from wild type (WT) with or without PF-012473224 and SCN10A$^{-/-}$ mice (WT: n=11 cells/5 mice; PF-01247324: n=11 cells/4 mice; SCN10A$^{-/-}$: n=10 cells/2 mice). Pharmacological inhibition or genetic knock-out of Na_V1.8 did not affect I_{Na} peak. One-way ANOVA with post-hoc Bonferroni's correction.

b Normalized original traces and mean data±SEM for steady-state inactivation (measured with the protocol in inset). The mean data for half-inactivation ($V_{1/2}$) are shown in the right panel. $V_{1/2}$ is derived from the curve by fitting it to standard Boltzmann equation: $h_\infty=1/\{1+\exp[(V_{1/2}-V)/K_\infty]\}$ (WT: n=9 cells/5 mice; PF-01247324: n=6 cells/3 mice; SCN10A$^{-/-}$: n=9 cells/2 mice). Pharmacological inhibition or genetic knock-out of Na_V1.8 did not affect steady-state inactivation. One-way ANOVA with post-hoc Bonferroni's correction.

c Mean data±SEM for I_{Na} intermediate inactivation. Increasing conditioning pulse duration (P_1) reduced I_{Na} amplitude assessed with a second pulse (P_2) consistent with entry of Na^+ channels into intermediate inactivation (protocol in inset). The mean data for plateau y_0 are shown in right panel (WT: n=12 cells/5 mice; PF-01247324: n=8 cells/5 mice; SCN10A$^{-/-}$: n=11 cells/3 mice). Pharmacological inhibition or genetic knock-out of Na_V1.8 did not affect intermediate inactivation. One-way ANOVA with post-hoc Bonferroni's correction.

d Mean data±SEM for recovery from inactivation. Increasing duration of the recovery interval between conditioning pulse (P1, causing I_{Na} inactivation) and test pulse (P2) results in an exponential increase in the

amplitude of I_{Na} upon P_2 consistent with I_{Na} recovery (protocol in inset). The mean data for the rate constant of recovery k_{rec} are shown in right panel. Pharmacological inhibition or genetic knock-out of $Na_V1.8$ did not affect recovery from inactivation (WT: n=8 cells/5 mice; PF-01247324: n=10 cells/5 mice; SCN10A$^{-/-}$: n=12 cells/3 mice). One-way ANOVA with post-hoc Bonferroni's correction.

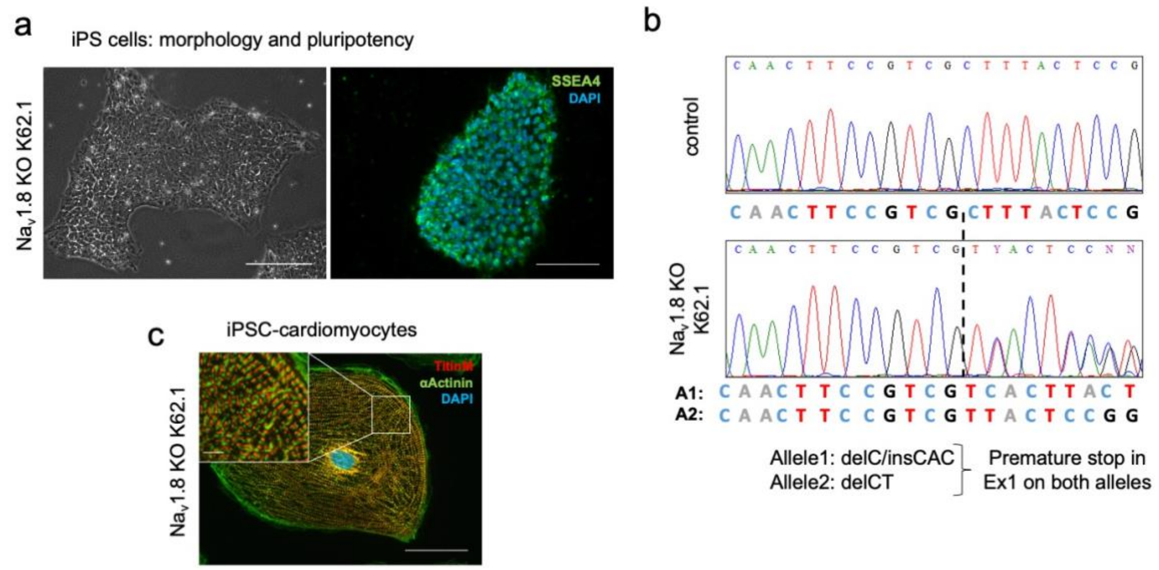

Supplementary Fig. 5: CRISPR/Cas9 generated homozygous *SCN10A* knock out iPSC-lines and cardiac differentiated iPSC-cardiomyocytes

a iPSCs show characteristic stem cell morphology and express the stem cell markers SSEA4 (depicted images are representative for both cell lines K62.1 and K62.4). Nuclei were stained with DAPI (blue). Scale bars: 200 µm for morphology and 100 µm for SSEA4. SSEA4 immunofluorescence staining was repeated for each cell line.

b Sanger Sequencing of generated KO iPSC-lines showing frameshifts in both alleles (A1, A2).

c iPSC-derived cardiomyocytes were stained for TTN (red) and α-actinin (green) and show a striated sarcomeric pattern. Nuclei were stained with DAPI (blue). Scale bars: 50 µM. TTN/ α-actinin immunofluorescence staining was repeated for each cardiac differentiation (3 differentiations used).

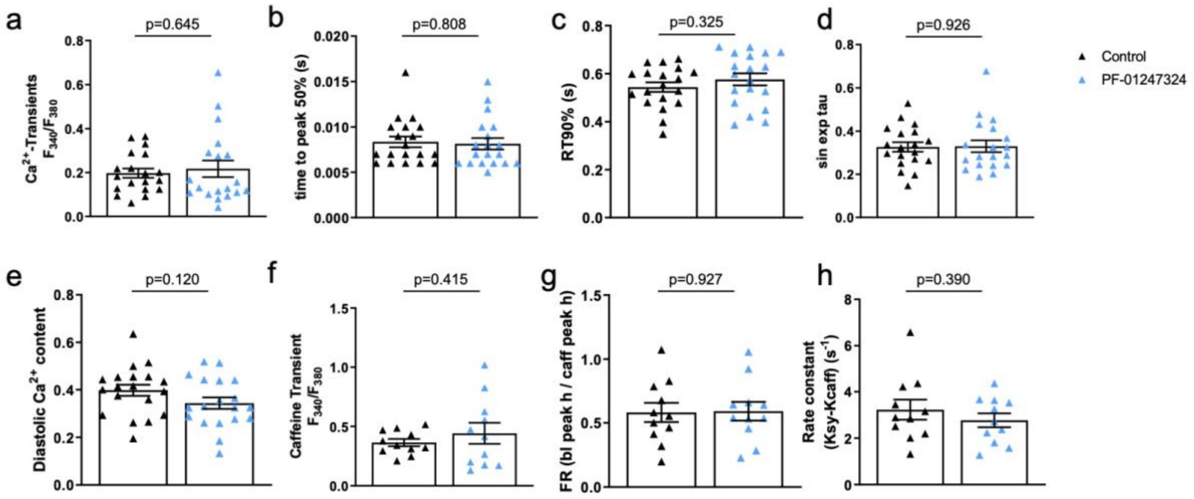

Supplementary Fig. 6: Influence of Na_V1.8 inhibition on Ca^{2+}-transient kinetics in Wild-Type (WT) mouse cardiomyocytes (extension of Fig. 3, main manuscript)

a Mean data±SEM showing no effect on Ca^{2+}-transient amplitude at 1 Hz (n=19 cells/5 mice).

b Mean data±SEM showing no effect on time to peak of the Ca^{2+}-transient at 1 Hz (n=19 cells/5 mice).

c Mean data±SEM showing no effect on Ca^{2+}-transient decay (90% of Ca^{2+}-removal) RT90 at 1 Hz (n=19 cells/5 mice).

d Mean data±SEM showing no effect on Ca^{2+}-transient decay time constant Sin exp tau at 1 Hz (n=19 cells/5 mice).

e Diastolic Ca^{2+} after addition of PF-01347324 was not differ compared to control at 1 Hz (n=19 cells/5 mice). Data are presented as mean values ± SEM.

f Mean data±SEM of caffeine-induced Ca^{2+}-transients was not affected by inhibition of Na_V1.8 (n=11 cells/5 mice).

g Ca^{2+}-fractional release (calculated from 1 Hz stimulated and caffeine induced transients) was not affected by inhibition of Na_V1.8 with PF-01247324 (n=11 cells/5 mice). Data are presented as mean values ± SEM.

h K_{sys}-K_{caff} as a measure for SERCA2a-activity was not affected by inhibition of Na_V1.8 with PF-01247324 (n=11 cells/5 mice). Data are presented as mean values ± SEM.

Data was analyzed by unpaired two-tailed Student's t-test Fig. S6 a-h).

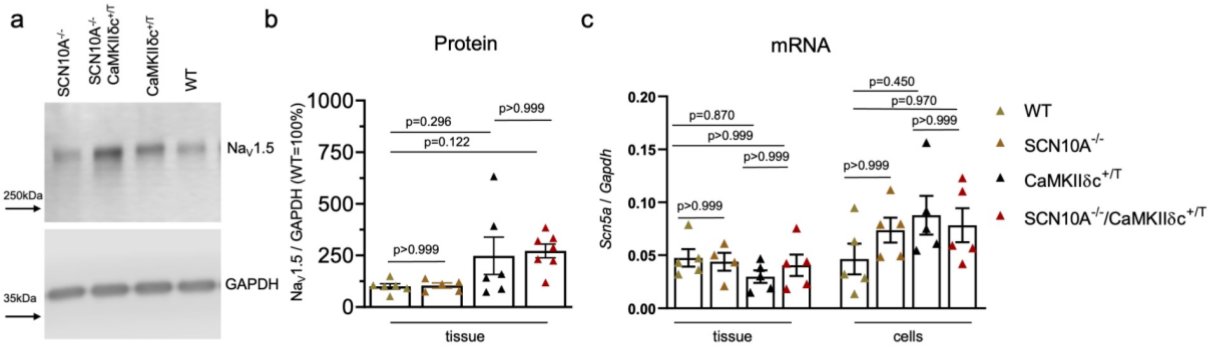

Supplementary Fig. 7: Influence of Na$_V$1.8 knock-out on Na$_V$1.5 protein and mRNA expression

a Original Western blot of Na$_V$1.5 in myocardial tissue of WT, SCN10A$^{-/-}$, CaMKIIδc$^{+/T}$ and SCN10A$^{-/-}$ / CaMKIIδc$^{+/T}$ mice.

b Western blot quantification of Na$_V$1.5 normalized to global protein loading. Na$_V$1.8 knock-out did not influence Na$_V$1.5 expression neither in SCN10A$^{-/-}$ vs. WT nor in SCN10A$^{-/-}$/CaMKIIδc$^{+/T}$ vs. CaMKIIδc$^{+/T}$ in mouse myocardium tissues (WT: n=6, SCN10A$^{-/-}$: n=5, CaMKIIδc$^{+/T}$: n=6, SCN10A$^{-/-}$/CaMKIIδc$^{+/T}$: n=7). One-way ANOVA with post-hoc Bonferroni's correction. Data are presented as mean values ± SEM.

c mRNA expression of *Scn5a* in myocardial tissue and isolated murine cardiomyocytes. *Scn10a* knock-out did not influence *Scn5a* mRNA expression neither in mouse myocardial tissue (WT: n=5, SCN10A$^{-/-}$: n=4, CaMKIIδc$^{+/T}$: n=5, SCN10A$^{-/-}$/CaMKIIδc$^{+/T}$: n=5) nor in cardiomyocytes (WT: n=5, SCN10A$^{-/-}$: n=5, CaMKIIδc$^{+/T}$: n=5, SCN10A$^{-/-}$/CaMKIIδc$^{+/T}$: n=5). One-way ANOVA with post-hoc Bonferroni's correction. Data are presented as mean values ± SEM.

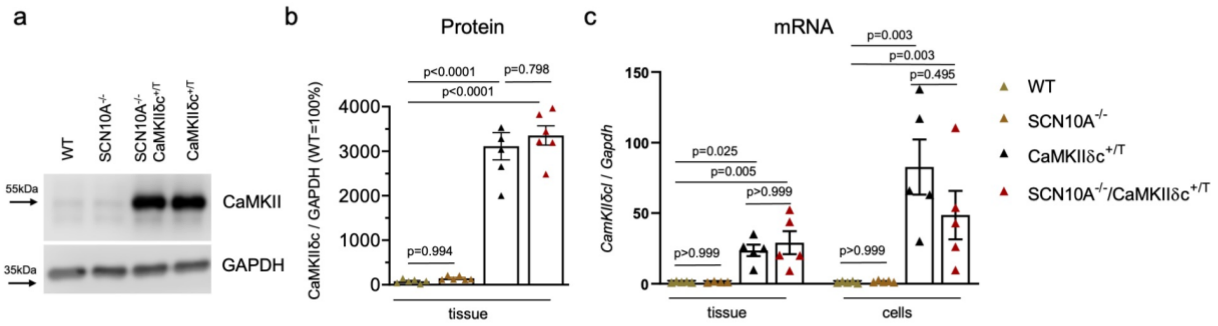

Supplementary Fig. 8: Influence of Na$_V$1.8 knock-out on CaMKIIδc protein and mRNA expression in mice

a Original western blot of CaMKIIδc in myocardial tissue of WT, SCN10A$^{-/-}$, CaMKIIδc$^{+/T}$ and SCN10A$^{-/-}$/ CaMKIIδc$^{+/T}$ mice.

b Western blot quantification of CaMKIIδc normalized to global protein loading. Significant upregulation of CaMKIIδc could be confirmed in the myocardium from CaMKIIδc$^{+/T}$ and SCN10A$^{-/-}$/CaMKIIδc$^{+/T}$ mice (WT: n=6, SCN10A$^{-/-}$: n=6, CaMKIIδc$^{+/T}$: n=6; SCN10A$^{-/-}$/CaMKIIδc$^{+/T}$: n=6) One-way ANOVA with post-hoc Bonferroni's correction. Data are presented as mean values ± SEM.

c mRNA expression of *CaMKIIδc* in myocardial tissue and isolated murine cardiomyocytes. Significant upregulation of *CaMKIIδc* could be confirmed in the myocardium (WT: n=5, SCN10A$^{-/-}$: n=4, CaMKIIδc$^{+/T}$: n=5, SCN10A$^{-/-}$/CaMKIIδc$^{+/T}$: n=5) and in the cardiomyocytes (WT: n=5, SCN10A$^{-/-}$: n=5, CaMKIIδc$^{+/T}$: n=5, SCN10A$^{-/-}$/CaMKIIδc$^{+/T}$: n=5) from CaMKIIδc$^{+/T}$ and SCN10A$^{-/-}$/CaMKIIδc$^{+/T}$. One-way ANOVA with post-hoc Bonferroni's correction. Data are presented as mean values ± SEM.

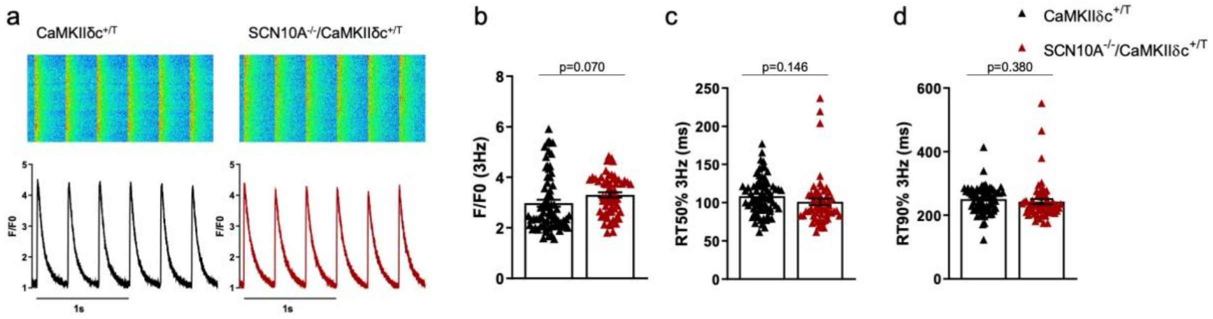

Supplementary Fig. 9: Ca^{2+}-transient amplitude and kinetics in cardiomyocytes from CaMKIIδc$^{+/T}$ and SCN10A$^{-/-}$/ CaMKIIδc$^{+/T}$ mice

a Original confocal line scans from Ca^{2+}-transient and Ca^{2+}-wave recordings and calculated intracellular Ca^{2+} displayed as F/F0 at 3 Hz (recorded Ca^{2+}-transients before stimulation was paused to record Ca^{2+}-waves).

b Mean data±SEM showing no effect of Nav1.8 knock-out on Ca^{2+}-transient amplitude in cardiomyocytes from CaMKIIδc$^{+/T}$ mice (F/F0 at 3 Hz) (CaMKIIδc$^{+/T}$: n=70 cells/9 mice, SCN10A$^{-/-}$/CaMKIIδc$^{+/T}$: n=58 cells/7 mice).

c Mean data±SEM showing no effect on Ca^{2+}-transient decay (50% of Ca^{2+}-removal) RT50 at 3 Hz (CaMKIIδc$^{+/T}$: n=70 cells/9 mice, SCN10A$^{-/-}$/CaMKIIδc$^{+/T}$: n=58 cells/7 mice).

d Mean data±SEM showing no effect on Ca^{2+}-transient decay (90% of Ca^{2+}-removal) RT90 at 3 Hz (CaMKIIδc$^{+/T}$: n=70 cells/9 mice; SCN10A$^{-/-}$/CaMKIIδc$^{+/T}$: n=58 cells/7 mice).

(Data was analyzed by unpaired two-tailed Student’s t-test Supplementary Fig. 9 b-d).

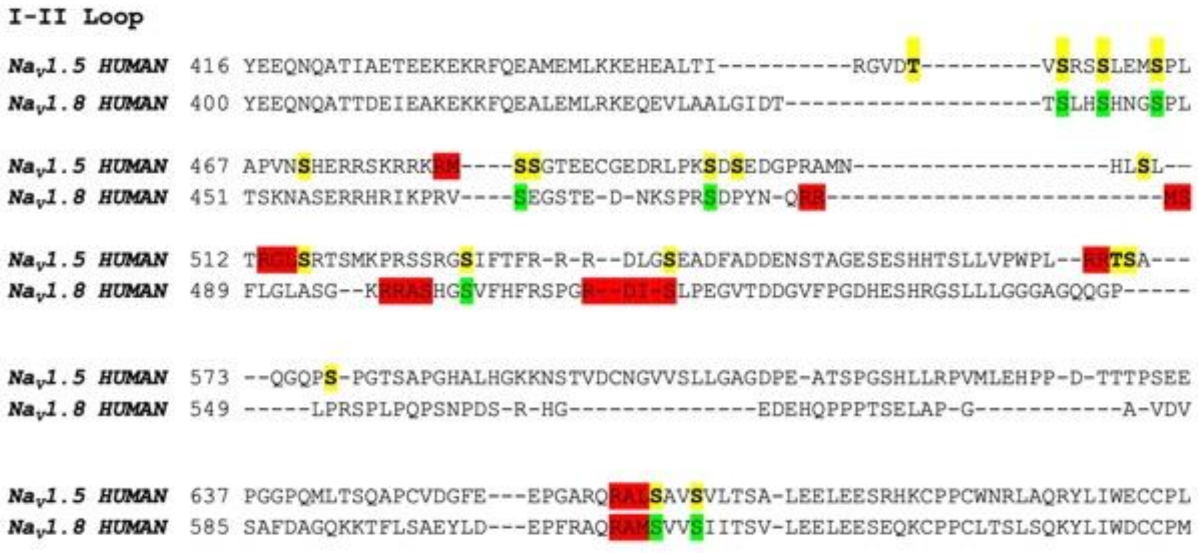

Supplementary Fig. 10: Amino acid sequence comparison of the I-II intracellular loop of $Na_V1.5$ and $Na_V1.8$

Amino acids sequences as published by Herren et al. J Proteome Re. 2015. CaMKII dependent phosphorylation sites from Herren et al are marked in yellow. Conserved Serin residues between $Na_V1.5$ and $Na_V1.8$ are marked in green. Serin residues with the CaMKII consensus motif are marked in red (serin/threonin residues of the consensus motif are marked in yellow).

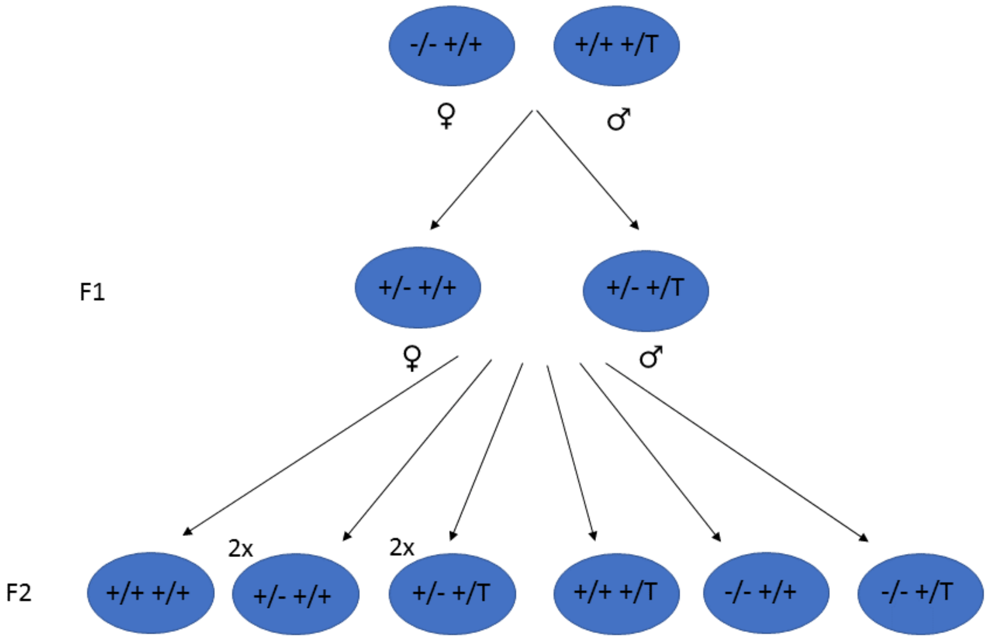

Supplementary Fig. 11: Scheme of crossbreeding of SCN10A$^{-/-}$ and CaMKIIδc$^{+/T}$ mice

Female SCN10A$^{-/-}$ were bred with male CaMKIIδc$^{+/T}$ mice, the F1 generation revealed heterozygote *SCN10A* knock-out and mice with transgenic or WT CaMKIIδc gene. The first genotype specification refers to the *SCN10A* gene, the second to CaMKIIδc. At the first position, -/- indicates homozygote knock-out of SCN10A, while -/+ only heterozygote and +/+ Wild-Type SCN10A. At the second position, +/+ indicates an unmodified CaMKIIδc gene, while +/T indicates heterozygote overexpression of CaMKIIδc.

Supplementary Tables:

Supplementary table 1: Action potential characteristics of ventricular cardiomyocytes from Wild-Type, SCN10A$^{-/-}$, CaMKIIδc$^{+/T}$ and SCN10A$^{-/-}$ / CaMKIIδc$^{+/T}$ mice (Supplement to Fig. 5):

Mean data±SEM of dv/dtmax, action-potential amplitude, resting membrane potential and action potential duration at 0.5, 1 and 2 Hz. Significance is indicated as vs. WT, One-Way-ANOVA with post-hoc Bonferroni-correction

	**Wild-Type**	**SCN10A $^{-/-}$**	**CaMKIIδc $^{+/T}$**	**SCN10A$^{-/-}$ / CaMKIIδc $^{+/T}$**
dv/dtmax (mV/ms)	96.5 ± 11.1	62.7 ± 10.4	67.4 ± 9.8	110.1 ± 16.4
AP amplitude (mV)	97.0 ± 4.2	83.3 ± 4.7	95.7 ± 3.7	99.4 ± 4.9
RM Potential (mV)	-66.9 ± 1.8	-68.4 ± 1.1	-65.6 ± 1.4	-67.8 ± 2.1
APD 90 0.5 Hz (ms)	43.6 ± 5.9	49.0 ± 8.9	142.0 ± 24.6 $^{p=0.004}$	124.6 ± 19.5 $^{p=0.010}$
APD 90 1 Hz (ms)	42.9 ± 5.8	49.6 ± 9.1	146.2 ± 31.0 $^{p=0.002}$	116.1 ± 15.7 $^{p=0.025}$
APD 90 2 Hz (ms)	48.4 ± 8.0	58.2 ± 11.0	132.8 ± 11.0 $^{p=0.011}$	134.3 ± 18.9 $^{p=0.004}$

Supplementary table 2: Baseline ECG-parameters of CaMKIIδc$^{+/T}$ and SCN10A$^{-/-}$/CaMKIIδc$^{+/T}$ mice (Supplement to Fig. 6): Mean data±SEM of HR=heart rate, QTcB (QT corrected by Bazett's formula) (CaMKIIδc$^{+/T}$: n=4 mice and SCN10A$^{-/-}$/CaMKIIδc$^{+/T}$: n=3 mice), *p<0.05 by unpaired two-tailed Students t-test.

	**CaMKIIδc $^{+/T}$ *(n=4)***	**SCN10A$^{-/-}$ / CaMKIIδc $^{+/T}$ *(n=3)***	**p-value**
HR (bpm)	500.0±30.9	523.8±13.8	0.56
RR interval (ms)	125.2±9.1	117.6±3.7	0.53
ST interval (ms)	37.0±1.7	36.3±1.4	0.77
QRS complex (ms)	21.0±1.7	16.5±2.0	0.08
PR interval (ms)	35.8±3.0	34.3±0.4	0.68
QT interval (ms)	51.3±1.0	47.6±1.2	0.07
QTcB interval (ms)	146.9±1.8	139.8±1.9	0.04 (*)

Supplementary table 3: Patient characteristics of human failing hearts used for experiments (14 patients) ICM: ischemic cardiomyopathy; EF: ejection fraction; LVEDD: Left ventricular end-diastolic diameter; ACE: ACE-inhibitor; β-B: β-blocker; DIU: diuretic; AMIO: Amiodarone (anti-arrhythmic); AT1: AT-1 receptor antagonists; MRA: mineralocorticoid receptor antagonist; CAT: catecholamine. Of note, clinical characteristics of 7 nonfailing heart donors are not available due to ethical reasons.

Patient data	mean±SEM
**Male sex (%)**	85.7
**age (years)**	48±5
**ICM (%)**	50
**Diabetes (%)**	21.4
**EF (%)**	23±1
**LVEDD (mm)**	61.7±3.2
**ACE (%)**	57.1
***β*-B (%)**	85.7
**DIU (%)**	78.5
**AMIO (%)**	57.1
**AT1 (%)**	21.4
**MRA (%)**	50
**CAT (%)**	21.4

Supplementary table 4: Primers used in this study

Primer	Forward (5´-3´)	Reverse (5´-3´)
Scn10a mouse	ATGGAGGTCAGCCAGGACTACA	CTGTGAGGTTGTCCGCACTGAA
Scn5a mouse	CAGACACTGATGACCAGGAAGAG	TCGGCTTCAGAGGATGTGGTCT
CamKIIδc mouse	GTGACACCTGAAGCCAAAGACC	CCTGTGCATCATGGAGGCAACA
Gapdh mouse	CATCACTGCCACCCAGAAGACTG	ATGCCAGTGAGCTTCCCGTTCAG
SCN10A human	TGGCAGATGACCTGGAAGAACC	CGATACGGTAGCAAGTCTTGCG
SCN5A human	TACACCTTTGAGTCTCTGGTCAAG	AATCACACTAAAGTCCAGCCAGTT
18S human	ACCCGTTGAACCCCATTCGTGA	GCCTCACTAAACCATCCAATCGG
