# Supplementary material for: Detrimental proarrhythmogenic interaction of Ca2+/calmodulin-dependent protein kinase II and NaV1.8 in heart failure
Source: Nat Commun. 2021 Nov 15;12:6586. doi: 10.1038/s41467-021-26690-1 (PMC8593192; doi:10.1038/s41467-021-26690-1)
Supplement: Supplementary file 3 — Reporting Summary [file 41467_2021_26690_MOESM3_ESM.pdf]

nature portfolio

Corresponding author(s): Prof. Dr. samuel Sossalla

Last updated by author(s): 09/15/2021

Reporting Summary

Nature Portfolio wishes to improve the reproducibility of the work that we publish. This form provides structure for consistency and transparency in reporting. For further information on Nature Portfolio policies, see our Editorial Policies and the Editorial Policy Checklist.

Statistics

For all statistical analyses, confirm that the following items are present in the figure legend, table legend, main text, or Methods section.

n/a	Confirmed	
☐	☒	The exact sample size (*n*) for each experimental group/condition, given as a discrete number and unit of measurement
☐	☒	A statement on whether measurements were taken from distinct samples or whether the same sample was measured repeatedly
☐	☒	The statistical test(s) used AND whether they are one- or two-sided<br>*Only common tests should be described solely by name; describe more complex techniques in the Methods section.*
☐	☒	A description of all covariates tested
☐	☒	A description of any assumptions or corrections, such as tests of normality and adjustment for multiple comparisons
☐	☒	A full description of the statistical parameters including central tendency (e.g. means) or other basic estimates (e.g. regression coefficient) AND variation (e.g. standard deviation) or associated estimates of uncertainty (e.g. confidence intervals)
☐	☒	For null hypothesis testing, the test statistic (e.g. *F*, *t*, *r*) with confidence intervals, effect sizes, degrees of freedom and *P* value noted<br>*Give P values as exact values whenever suitable.*
☒	☐	For Bayesian analysis, information on the choice of priors and Markov chain Monte Carlo settings
☒	☐	For hierarchical and complex designs, identification of the appropriate level for tests and full reporting of outcomes
☒	☐	Estimates of effect sizes (e.g. Cohen's *d*, Pearson's *r*), indicating how they were calculated

Our web collection on statistics for biologists contains articles on many of the points above.

Software and code

Policy information about availability of computer code

Data collection	• Confocal images and line scans were collected using ZEN 2009 (version 6.0) or Axiovision software 4.8<br>• Patch Clamp data were collected using Patchmaster 2.0 (HEKA Elektronik)<br>• Ca2+ transients were collected with the software IONWizardR version 6.4 (ION OPTIX Corp.).<br>• Echocardiography was performed using a VS-VEVO 660/230 (Visualsonics).
Data analysis	• Analysis of the Western Blot bands and the histological stainings was performed with Image J 1.51j8 (NIH, USA).<br>• Ca2+ sparks were analysed with the SparkMaster plugin 2006 for ImageJ (PMID: 17376815).<br>• Ca2+ transients were analyzed with the software IONWizardR version 6.4 (ION OPTIX Corp.).<br>• Membrane potential recordings were analyzed using LabChart 7.37 (ADInstruments)<br>• ECG recordings were analysed with Ponemah Physiology Platform 6.3<br>• Data analysis was performed and graphs were plotted with GraphPad Prism version 8.0 GraphPad Software<br>• Western Blots were analysed using Image Studio Lite Ver 5.2

For manuscripts utilizing custom algorithms or software that are central to the research but not yet described in published literature, software must be made available to editors and reviewers. We strongly encourage code deposition in a community repository (e.g. GitHub). See the Nature Portfolio guidelines for submitting code & software for further information.

Data

Policy information about availability of data

All manuscripts must include a data availability statement. This statement should provide the following information, where applicable:

- Accession codes, unique identifiers, or web links for publicly available datasets
- A description of any restrictions on data availability
- For clinical datasets or third party data, please ensure that the statement adheres to our policy

All data generated or analyzed during this study are available within the Article and its Supplementary Information. All raw data supporting the findings from this study are available from the corresponding author upon reasonable request. Source data are provided with this paper. Any remaining raw data will be available from the corresponding author upon reasonable request.

Field-specific reporting

Please select the one below that is the best fit for your research. If you are not sure, read the appropriate sections before making your selection.

☒ Life sciences ☐ Behavioural & social sciences ☐ Ecological, evolutionary & environmental sciences

For a reference copy of the document with all sections, see nature.com/documents/nr-reporting-summary-flat.pdf

Life sciences study design

All studies must disclose on these points even when the disclosure is negative.

Sample size	Sample sizes were determined based on our extensive past experience with these types of experiments (PMID: 29931291, PMID: 30378291, PMID: 21251589). All experiments were conducted with at least three indenpendent experiments and multiple independent biological replicates. For electrophysiology experiments comparing multiple cells from different mice/patients were required and we generally aimed for Ns of >10 to control adequately for inter-cell variability, following our experience and prior reports in the literature. For biochemical analyses, we required at least 4-7 independent experiments with each group. Adequacy of sample size was confirmed on the basis that statistically non-significant differences were of an order that suggested lack of biological significance.
Data exclusions	There was no exclusion of experiments.
Replication	All experimental series were repeated in different experiments and separate days. Each N in the paper represents a measurement on a separate cell or preparation; repeated measurements were not obtained. Replication was ensured by performing a sufficient number of independent measurements, each on separate samples or subjects (cells), to ensure replication of the biological phenomenon being measured.
Randomization	Randomization was not possible in the studies in crossbred mice because the sample identity was determined by its source. For the studies involving pharmacological interventions, no formal randomization was performed but group allocation was arbitrary and not related to any properties of samples or subjects. The sequence of the pharmacological interventions used in an individual subject was changed from animal to animal, so that there was no time factor after cell isolation in the whole data set.
Blinding	Blinded analysis was performed for Ca2+-spark analysis and in-vivo echocardiography/ecg-analysis. Data collection of Ca2+-sparks was not blinded as the investigator had to prepare the drug used for the pharmacological intervention. For in-vivo echocardiography and ecg-analysis the examiner was also blinded for data collection. Survival data of individual mice was documented by the staff of the local animal facility which was blinded to the genotype of the animals. Ca2+-transient data collection and analysis was not blinded as analysis was performed automatically by the ION Wizard software. Patch-Clamp, western-blot and mRNA experiments were also automatically analysed by the software used for analysis.

Reporting for specific materials, systems and methods

We require information from authors about some types of materials, experimental systems and methods used in many studies. Here, indicate whether each material, system or method listed is relevant to your study. If you are not sure if a list item applies to your research, read the appropriate section before selecting a response.

Materials & experimental systems

n/a	Involved in the study
	☒ Antibodies
☒	☐ Eukaryotic cell lines
☒	☐ Palaeontology and archaeology
	☒ Animals and other organisms
	☒ Human research participants
☒	☐ Clinical data
☒	☐ Dual use research of concern

Methods

n/a	Involved in the study
☒	☐ ChIP-seq
☒	☐ Flow cytometry
☒	☐ MRI-based neuroimaging

Antibodies

Antibodies used

• Rabbit polyclonal anti-CaMKIIδ: 1: 100 dilution for IF, 3 µg/500 µg of protein for Co-IP; gift from D. M. Bers, University of California Davis, USA).
• Rabbit polyclonal anti-CaMKIIδ: 1:5000 dilution for WB, Thermo Scientific, Catalog # PA5-22168,
• anti-control IgG: (3 µg/500 µg protein for Co-IP; Santa Cruz, Catalog # sc-2027,
• Mouse monoclonal anti-NaV1.8: 1:50 dilution for IF, Clone name S134-12, LSBio, Catalog # LS-C109037,
• Rabbit polyclonal anti-NaV1.8: 1:1000 dilution for WB, Alomone labs, Catalog # ASC-016,
• Mouse monoclonal anti-GAPDH: 1:20000 dilution for WB, Clone name 6C5, Biotrend, Catalog # BTMC-A437-9,
• Rabbit polyclonal anti-NaV1.5: 1:2000 dilution for WB, Alomone labs, Catalog # ASC-005,
• Mouse monoclonal anti-SSEA4: 1:200 dilution for IF, Clone name MC813, Abcam, Catalog # ab16287,
• Mouse anti-α-actinin: 1:750 dilution for IF, Sigma, Catalog # A7811,
• Rabbit anti-Titin-M8/M9: 1:750 dilution for IF, MyoMedix, Catalog # TTN-10,
• Alexa Fluor-488 goat anti-mouse: 1:200 dilution for IF, Invitrogen, Catalog # A-11029,
• Alexa Fluor-555 goat anti-rabbit: 1:200 dilution for IF, Invitrogen, Catalog # A-21428,
• Alexa Fluor-488 donkey anti-mouse IgG: 1:1000 dilution for IF, Invitrogen, Catalog # A-21202
• Alexa Fluor-555 donkey anti-rabbit IgG: 1:750 dilution for IF, Invitrogen, Catalog # A-31572
• HRP-conjugated goat anti-rabbit: 1:20000 dilution for WB, Jackson Immunoresearch, Catalog # 111-035-144
• HRP-conjugated goat anti-mouse 1:20000 dilution for WB, Jackson Immunoresearch Catalog # 115-035-062

Validation

• Rabbit polyclonal anti-CaMKIIδ: gift from D. M. Bers, University of California Davis, USA).
Custom made anti-CaMKIIδ antibody was validated in Huke and Bers, J Mol Cell Cardiol. 2007 (PMID: 17239900)

• Rabbit polyclonal anti-CaMKIIδ: Thermo Scientific, Catalog # PA5-22168
Manufacture's website: https://www.thermofisher.com/antibody/product/CaMKII-delta-Antibody-Polyclonal/PA5-22168.
Citation: Cardiovasc Diabetol. 14;17(1):89 (2018) (PMID: 29903013).

• anti-control IgG: (3 µg/500 µg protein for Co-IP; Santa Cruz, Catalog # sc-2027
Manufacture's website: https://datasheets.scbt.com/sc-2027.pdf
Citation: EMBO J. 20: 1739-1753. (2001) (PMID: 11285237)

• Mouse monoclonal anti-NaV1.8: 1:50 dilution for IF, LSBio, Clone name S134-12 Catalog # LS-C109037,
Manufacture's website: https://www.lsbio.com/antibodies/scn10a-antibody-nav1.8-antibody-aa1724-1956-clone-s134-12-icc-ihc-wb-western-ls-c109037/111165.
Citation: Cardiovasc Res 1;114(13):1728-1737 (2018) (PMID: 29931291).

• Rabbit polyclonal anti-NaV1.8: 1:1000 dilution for WB, Alomone labs, Catalog # ASC-016,
Manufacture's website: https://www.alomone.com/p/anti-nav1-8-2/ASC-016.
Citation: J Neurosci 21;35(3):1125-35 (2015) (PMID: 25609627).

• Mouse monoclonal anti-GAPDH: 1:20000 dilution for WB, Biotrend, Clone name 6C5, Catalog # BTMC-A437-9,
Manufacture's website: https://www.biotrend.com/en/other-products-186/mouse-monoclonal-anti-rabbit-gapdh-1206505595.html
Citation: Cardiovasc Res 1;114(13):1728-1737 (2018) (PMID: 29931291).

• Rabbit polyclonal anti-NaV1.5: 1:2000 dilution for WB, Alomone labs, Catalog # ASC-005,
Manufacture's website: https://www.alomone.com/p/anti-nav1-5/ASC-005?gclid=EAIaIQobChMI3JfO5Mj18QIVCqh3Ch3wtwL6EAAYASAAEgLqHfD_BwE
Citation: J Biol Chem 1;294(44):16123-16140 (2019) (PMID: 31511323)

• Mouse monoclonal anti-SSEA4: 1:200 dilution for IF, Abcam, Clone name MC813, Catalog # ab16287,
Manufacture's website: https://www.abcam.com/ssea4-antibody-mc813-70-ab16287.html;
Citation: EMBO Mol Med 12:e12146 (2020) (PMID: 32840960)

• Mouse anti-α-actinin: 1:750 dilution for IF, Sigma, Catalog # A7811,

Manufacture's website: https://www.sigmaaldrich.com/DE/en/product/sigma/a7811?context=product
Citation: Nat Commun 10, 4671 (2019) (PMID: 31604922)

• Rabbit anti-Titin-M8/M9: 1:750 dilution for IF, MyoMedix, Catalog # TTN-10,
Manufacture's website: http://www.myomedix.com/seiten/products.htm;
Citation: Stem Cell Reports 12(5):1145-1158 (2019) (PMID: 30956114)

• Alexa Fluor-488 goat anti-mouse: 1:200 dilution for IF, Invitrogen, Catalog # A-11029,
Manufacture's website: https://www.thermofisher.com/antibody/product/Goat-anti-Mouse-IgG-H-L-Highly-Cross-Adsorbed-Secondary-Antibody-Polyclonal/A-11029
Citation: Nat Commun 5;10(1):3991 (2019) (PMID: 31488816).

• Alexa Fluor-555 goat anti-rabbit: 1:200 dilution for IF, Invitrogen, Catalog # A-21428,
Manufacture's website: https://www.thermofisher.com/antibody/product/Goat-anti-Rabbit-IgG-H-L-Cross-Adsorbed-Secondary-Antibody-Polyclonal/A-21428.
Citation: Cancer Res 1;78(17):5107-5123 (2018) (PMID: 29997232).

• Alexa Fluor-488 donkey anti-mouse IgG: 1:1000 dilution for IF, Invitrogen, Catalog # A-21202
Manufacture's website: https://www.thermofisher.com/antibody/product/Donkey-anti-Mouse-IgG-H-L-Highly-Cross-Adsorbed-Secondary-Antibody-Polyclonal/A-21202;
Citation: Stem Cell Res. 38:101461 (2019) (PMID: 31132580)

• Alexa Fluor-555 donkey anti-rabbit IgG: 1:750 dilution for IF, Invitrogen, Catalog # A-31572
Manufacture's website: https://www.thermofisher.com/antibody/product/Donkey-anti-Rabbit-IgG-H-L-Highly-Cross-Adsorbed-Secondary-Antibody-Polyclonal/A-31572;
Citation: Cell Stem Cell. 23(6):820-832.e9. (2018) (PMID: 30416070)

• HRP-conjugated goat anti-rabbit: 1:20000 dilution for WB, Jackson Immunoresearch, Catalog # 111-035-144
Manufacture's website: https://www.jacksonimmuno.com/catalog/products/111-035-144.
Citation: Sci Rep 11:11185 (2021) (PMID: 34045646).

• HRP-conjugated goat anti-mouse 1:20000 dilution for WB, Jackson Immunoresearch Catalog # 115-035-062
Manufacture's website: https://www.jacksonimmuno.com/catalog/products/115-035-062.
Citation: Nat Commun 8;12(1):848 (2021) (PMID: 33558493).

Animals and other organisms

Policy information about studies involving animals; ARRIVE guidelines recommended for reporting animal research

Laboratory animals	To generate SCN10A-/-/CaMKIIδc+/T mouse model we crossbred CaMKIIδC+/T mice from a Black-Swiss strain with NaV1.8 knockout mice (SCN10A-/-) from a C57BL/6 strain. 8 weeks old male CaMKIIδC+/T mice were mated with 8 weeks old female SCN10A-/- mice (C57BL/6).<br>For Fig. 2 and 3 12 week old male and female mice were used from the Black-Swiss strain.<br>For Fig. 4 and 5 and all supplementary figures 12 week old male and female mice from the crossbreeding were used. For in-vivo telemetry recordings male mice at an age of 8 weeks were used.<br>The breeding rooms were maintained at 20-22°C with 50-60% humidity. Mice were housed in a room with a 12-hour light/dark cycle with ad libitum access to water and food.
Wild animals	The study did not involve wild animals
Field-collected samples	The study did not involve field-collected samples
Ethics oversight	The study was approved by the local ethics committee of the University Medicine of Goettingen and the public authority on animal welfare.

Note that full information on the approval of the study protocol must also be provided in the manuscript.

Human research participants

Policy information about studies involving human research participants

Population characteristics	Patient characteristics of all end-stage heart failure patients donating left ventricular myocardium after undergoing heart transplantation are described in Supplementary table 1.<br><br>iPSCs were generated from a control donor, healthy, female, 52 years.
Recruitment	We obtained left ventricular tissues from explanted hearts of patients with end-stage HF (NYHA HF classification IV) who

	were undergoing heart transplantation. All patients undergoing heart transplantation were asked to participate in this study. Patients gave informed consent for tissue donation before surgery. Hearts from patients with rare diseases or congenital heart defects were excluded from the experiments.
Ethics oversight	All experiments of the study were approved by the local ethics committee of the University Medicine of Goettingen (Ethik-Kommission der Universitätsmedizin Göttingen, Prof. Jürgen Brockmöller).

Note that full information on the approval of the study protocol must also be provided in the manuscript.
